# Coherence-Gated Sensorless Adaptive Optics Multiphoton Retinal Imaging

**DOI:** 10.1038/srep32223

**Published:** 2016-09-07

**Authors:** Michelle Cua, Daniel J. Wahl, Yuan Zhao, Sujin Lee, Stefano Bonora, Robert J. Zawadzki, Yifan Jian, Marinko V. Sarunic

**Affiliations:** 1 School of Engineering Science, Simon Fraser University, Burnaby, BC V5A 1S6 Canada; 2 CNR-Institute for Photonics and Nanotechnology, Via Trasea 7, 35131, Padova, Italy; 3 UC Davis RISE Small Animal Ocular Imaging Facility, Department of Cell Biology and Human Anatomy, University of California Davis, Davis, CA 95616, USA; 4 Vision Science and Advanced Retinal Imaging laboratory (VSRI), Department of Ophthalmology & Vision Science, University of California Davis, Sacramento, CA 95817 USA

## Abstract

Multiphoton microscopy enables imaging deep into scattering tissues. The efficient generation of non-linear optical effects is related to both the pulse duration (typically on the order of femtoseconds) and the size of the focused spot. Aberrations introduced by refractive index inhomogeneity in the sample distort the wavefront and enlarge the focal spot, which reduces the multiphoton signal. Traditional approaches to adaptive optics wavefront correction are not effective in thick or multi-layered scattering media. In this report, we present sensorless adaptive optics (SAO) using low-coherence interferometric detection of the excitation light for depth-resolved aberration correction of two-photon excited fluorescence (TPEF) in biological tissue. We demonstrate coherence-gated SAO TPEF using a transmissive multi-actuator adaptive lens for *in vivo* imaging in a mouse retina. This configuration has significant potential for reducing the laser power required for adaptive optics multiphoton imaging, and for facilitating integration with existing systems.

Multiphoton microscopy (MPM) is an increasingly common imaging modality that is used to obtain 3D images from biological specimen with molecule-specific contrast. The use of MPM for *in vivo* microscopy has multiple potential benefits over single photon microscopy^1^. Unlike single photon processes, MPM techniques such as two-photon excited fluorescence (TPEF) only occur at a narrow axial range around the focal point where the irradiance is highest, thereby providing an optical sectioning effect. Compared to conventional microscopy with single photon excitation fluorescence, MPM commonly uses light at longer wavelengths, in the near infrared (NIR), where tissue scattering and absorption are lower. The use of NIR light is particularly attractive for imaging the retina since the photoreceptor cells contain visual pigments that are sensitive to visible wavelengths. The main disadvantage of MPM imaging in ocular tissues is the high pulse energy required to elicit the non-linear effects. Minimizing the incident exposure energy is paramount for non-invasive imaging, in particular for the delicate tissues of the retina. Although MPM is relatively unaffected by low levels of out-of-focus scattering, wavefront aberrations from the sample and optical path broaden and distort the focal spot. The MPM signal is quartically proportional to the focused spot size^2^; hence, significant improvements in the signal-to-noise ratio can be achieved through wavefront shaping to approach the diffraction-limited focus with a large numerical aperture, in particular for applications such as retinal imaging.

The adaptive optics (AO) techniques that have been developed for astronomical telescopes can also be applied to microscopy and ocular imaging to correct for refractive errors and enable diffraction-limited focusing. Conventional AO systems developed for retinal imaging use a Hartmann-Shack Wavefront Sensor (HS-WFS) to detect the wavefront aberrations and, in a closed feedback loop control, guide the shape of an adaptive element, such as a deformable mirror, to correct the wavefront aberrations^3^,^4^. The HS-WFS is sensitive to back reflections, and as a result, many conventional AO systems use curved mirrors instead of lenses, and long focal lengths to minimize the off-axis aberrations^5^,^6^. Furthermore, the use of a wavefront sensor places significant design constraints on the system, as it requires optical conjugation of the pupil plane of the sample, the deformable element, and the HS-WFS, which can introduce non-common path errors in particular between the HS-WFS and the deformable mirror^7^. Another limitation of the HS-WFS is that it is only functional when there is a single scattering plane in the sample; thick tissue samples or multi-layered samples impede the ability to measure the wavefront. For mouse eyes, the presence of multiple reflective surfaces, which can result in double spot formation on the WFS^8^ or broad, unfocused spots (albino mice)^9^, makes the use of sensor-based AO approaches challenging. Although HS-WFS AO has been successfully performed in living mouse retina^10^,^11^,^12^,^13^,^14^,^15^, it was constrained by high-NA low depth-of-focus imaging to reduce the reflections from other surfaces, and aberration correction was only performed on the outer, most reflective, layer of the retina^11^.

Sensorless Adaptive Optics (SAO) techniques have been developed to overcome the limitations of the HS-WFS. Image-based SAO methods that optimize the shape of the deformable element to correct the wavefront based on indirect measurements have been successfully used in microscopy to perform aberration correction^16^,^17^,^18^,^19^. An SAO confocal Scanning Laser Ophthalmoscope (cSLO) has been reported in the literature for human retinal imaging, with performance demonstrated to be comparable to HS-WFS-based AO systems^20^. We have reported on an SAO technique for high-resolution retinal imaging with Optical Coherence Tomography (OCT) in small animals and humans^9^,^21^,^22^,^23^, as well as an SAO biomicroscope for imaging fluorescently labeled cells in the mouse retina^24^. A contrast-based SAO ophthalmoscope was also demonstrated for imaging of human and animal eyes^25^. The SAO cSLO and OCT systems completed the wavefront-correction algorithm in a time on the order of seconds and showed excellent results. An SAO approach has also been reported in the literature using TPEF images in mouse retina to guide aberration correction^26^; however, this system required 6–7 minutes to perform a single optimization using high power laser excitation.

For *in vivo* retinal imaging, the combination of AO with MPM should be fast (on the order of seconds), and should minimize the high-energy laser exposure on the delicate light-sensitive tissue. We present a novel depth-resolved SAO for MPM that is well suited to retinal imaging applications. We combined MPM imaging with depth-resolved SAO using the same light source, but separate detection systems. The light used for MPM microscopy typically consists of a train of pulses that are on the order of femtoseconds in duration. The femtosecond-pulsed light source can be selected to have adequate bandwidth (tens of nanometers) for low coherence interferometry detection with an axial resolution on the order of microns^27^. Low coherence interferometry is the basis of OCT, and enables the acquisition of coherence-gated cross-sectional images of a sample. Due to the high sensitivity of the OCT detection, a cross-sectional profile of the sample can be visualized along a depth that is much larger than the Rayleigh range of the focused beam. The OCT images thus constitute a coherence-gated, depth-resolved signal that can be used for image-guided SAO aberration correction of the excitation beam in the sample. The optimization can be performed at lower power since the back-scattered light used for the OCT detection is a single-photon process. Following the aberration correction, the excitation laser intensity can be increased to perform the MPM imaging with a separate dedicated highly sensitive photodetector. Both the MPM and the OCT-guided SAO share the same source and sample arm delivery optics to ensure exact co-registration of the images during acquisition.

We present results demonstrating the improvement in the MPM signal after OCT-guided SAO aberration correction on a phantom and for *in vivo* mouse retina imaging. The image acquisition was performed with a home-built multiphoton microscope using a novel transmissive multi-actuator adaptive lens (MAL) as the deformable element^22^.

## Methods

### Multiphoton Imaging System with OCT

**Figure 1:**
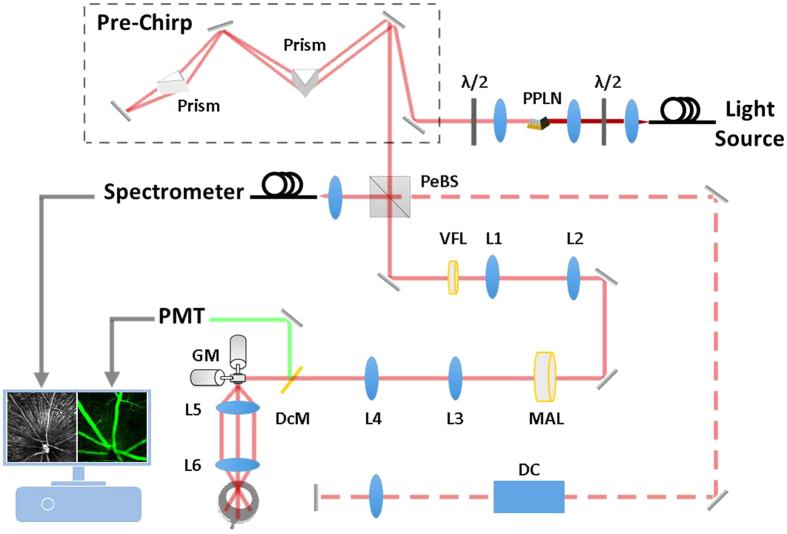
Schematic of the proposed wavefront sensorless adaptive optics multiphoton microscopy (SAO-MPM) system with optical coherence tomography (OCT) guided aberration correction. A second-harmonic-generating module was used to frequency-double the 1560 nm light source. PPLN, periodically-poled lithium niobate crystal. PBS, pellicle beam splitter; VFL, variable focus lens; MAL, multi-actuator adaptive lens; DcM, dichroic mirror; GM, galvanometer-scanning mirrors; PMT, photo-multiplier tube; DC, dispersion compensation; L1, 60 mm; L2, 300 mm; L3, 100 mm; L4, 400 mm; L5, 50 mm; L6, 17 mm; Reference arm denoted as a dashed line.

A schematic of our experimental imaging system is presented in Fig. 1. The source light was a 1560 nm femtosecond laser (Menlo Systems, Germany) with 120 nm bandwidth and 47 fs pulse duration at the laser output. The output of the laser was focused through a periodically-poled lithium niobate (PPLN) crystal and frequency doubled to a spectrum with centre wavelength of ~800 nm, which was used as the MPM excitation source. The frequency-doubled light had a non-Gaussian spectrum with an approximate full width half maximum bandwidth of 18 nm, corresponding to an estimated axial resolution of ~12 μm for the OCT sub-system. The excitation light was directed through a dispersion-compensating prism pair (DCP) to compensate for the group delay dispersion from the optical elements and provide an approximately transform-limited pulse duration at the sample in order to maximize the MPM signal. The dispersion pre-compensation was adjustable to accommodate different samples, for example the mouse eye. After pulse compression, the light was split by a pellicle beam splitter (PBS), with 95% of the power directed towards the sample, and 5% of the power directed towards a reference arm used for interferometry. The sample arm consisted of: galvanometer-scanning mirrors (GM) to scan the light across the surface of the sample, a MAL to correct the wavefront aberrations, a variable focus Lens (VFL, ARCTIC 316-AR850, Lyon, France) to control the focal plane in the sample, and three telescopes to relay the conjugate plane from the VFL to MAL to GM, and finally to the pupil of the mouse eye. Note that although the GM is schematically presented as a single element for clarity, two-dimensional scanning was accomplished using an XY mounted pair of galvanometer mounted mirrors with a clear aperture of 3 mm.

Two-photon excited fluorescence emission from the sample was de-scanned by the GM and then reflected by the dichroic mirror (DcM) to the photo-multiplier tube (PMT) detector. A short-pass filter, lens, and aperture were placed prior to the PMT to reject residual excitation light and stray reflections.

The back-scattered excitation light was de-scanned at the GM, transmitted by the dichroic mirror, and recombined with the reference arm light at the beam splitter. The recombined light was fiber coupled to a spectrometer (Bioptigen, Inc., Durham, NC). The sample and reference light generated an interference pattern on the spectrometer, which was processed into cross-sectional images using a custom GPU-accelerated program^28^,^29^,^30^. Our OCT engine was configured to acquire A-scans at a rate of 50 kHz. For the optimization, the OCT volume acquisition size (1024 axial × 200 lateral × 200 elevation voxels) was selected to obtain a balance between sampling density and acquisition speed with consideration to the limitations of the speed of the galvanometer-scanning mirrors. These acquisition parameters resulted in an acquisition rate of 200 B-scan frames per second, equivalent to 1 volume per second with processing and display in real-time. The acquisition of the OCT A-scans was synchronized to the digitization of the PMT, which ensured that both OCT and MPM images were perfectly registered. After OCT-guided SAO optimization, we switched our imaging system to TPEF imaging mode, where we acquired TPEF images only at 10 frames per second (200 × 200), with 50 frames of images streamed to disk per acquisition. The TPEF images were rigidly registered and averaged for presentation in Matlab (Mathworks, Natick, MA).

*In vivo* imaging of mouse retinal vasculature was also demonstrated using the OCT-guided SAO TPEF. Mice were anesthetized using ketamine (100 mg/kg of body weight) and dexmedetomidine (0.1 mg/kg of body weight), and subcutaneously injected with 100 μL of 100 mg/mL fluorescein. Prior to imaging experiments, the eyes were dilated with 1% Tropicamide, then topical anesthetic (Alcaine, 0.5%) and artificial tear gel (Alcon, Fort Worth, TX) was applied. Lastly, a rigid contact lens was placed on the mouse eye to prevent the cornea from drying^31^. The OCT images were used to guide the alignment of the mouse eye. The alignment was initially performed using a wide-scan to visualize the location of the dilated iris, and completed based on the OCT images of the mouse retina. The position of the focus within the axial extent of the retina was controlled by the VFL and was observed as a bright layer in the OCT B-scan that changed in depth position^32^. Using the real-time OCT cross-sectional image feedback, the focus was placed on the depth layer of interest. Aberration correction was performed using the *en face* OCT images extracted from the volume at a depth corresponding to this focal plane. All imaging experiments were carried out in accordance to the protocols approved by the University Animal Care Committee at Simon Fraser University. The numerical aperture of the imaging beam was calculated to be 0.21. The estimated focal spot size, assuming a Gaussian beam profile for the incident beam, was ~1.5 μm (Gaussian waist). This corresponded to an estimated Rayleigh range for the imaging beam of ~9.5 μm. The laser power incident on the mouse eye was limited to a maximum of ~10 mW for both the OCT-guided SAO optimization and MPM imaging.

### OCT-guided SAO

The SAO optimization algorithm was modified from our previous reports on imaging mice and humans^9^,^21^,^24^. Briefly, the SAO optimization process was initialized via manual selection of the depth plane that corresponded to the desired location of focus within the sample. This depth plane was selected using the cross-sectional information from the B-scan images acquired using the OCT sub-system. An *en face* image was generated from the user-selected depth region within the OCT volume by maximum intensity projection (MIP); the sharpness of this 2D *en face* image, calculated using Equation 1, was used as the merit function for the SAO optimization^33^:


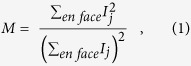


where *I*_*j*_ is the intensity value of the *j*-th pixel in the OCT *en face* image, and the summation is performed on the entire OCT *en face* image.

The wavefront aberrations were represented using a set of orthogonal Zernike polynomials, which allowed us to optimize each Zernike mode independently. The MAL is capable of correcting aberrations up to 4th order Zernike polynomials, however aberration correction was restricted to the second and third order Zernike polynomials (defocus, astigmatisms and comas) in consideration of the limited stroke of the MAL. We first optimized for defocus (Z = 4), followed by two astigmatisms (Z = 3, 5) and then the two comas (Z = 7, 8). For each Zernike mode, the optimization was performed by first acquiring an OCT volume for 11 different coefficients applied to the MAL. For each coefficient value, an *en face* image was extracted, and the coefficient that produced the sharpest image was selected as the optimized value. The optimization of the next Zernike mode continued in a hill-climbing fashion, using the combination of the previously optimized modes as a starting point. Based on the acquisition parameters of the OCT system, the optimization was completed in ~60 s.

## Results

**Figure 2:**
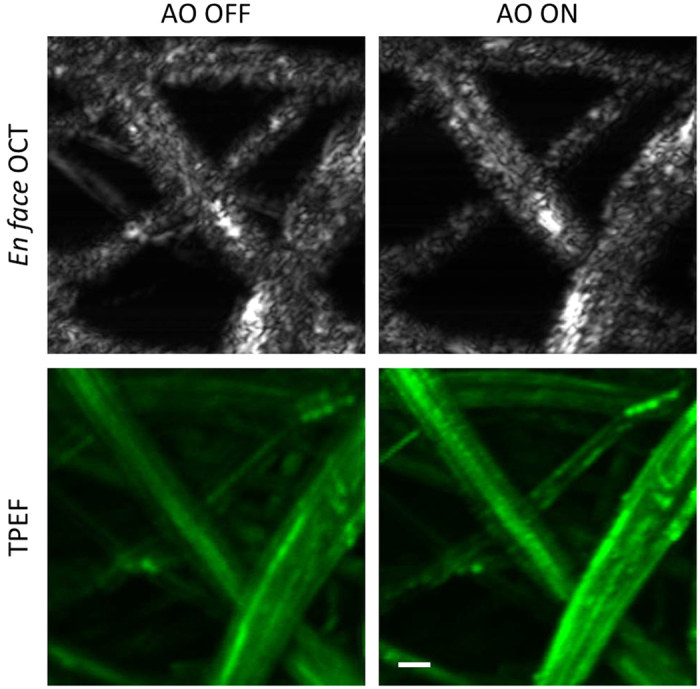
Representative images of MPM with OCT-guided SAO aberration correction. The TPEF images in this figure are the average of 50 rigidly registered TPEF frames. Scale bars: 70 μm.

The impact of OCT-guided SAO wavefront correction on the quality of the MPM images was first demonstrated on an imaging phantom. Lens paper stained with a fluorescein derivative fluorescent dye (excitable by a two-photon process at the ~800 nm incident wavelength) was covered with a layer of clear epoxy and sealed with a microscope coverslip. An aspheric lens (with focal length of 2.97 mm) was used as an objective lens to focus the beam on the phantom. The purpose of the epoxy and coverslip was to introduce distortions for the demonstration of aberration correction with the OCT-guided SAO. The fibers in the lens paper provided a structural back-scattering image for the OCT, with the intention of improving the TPEF signal from the dye, which represented the signal of interest in the phantom. Using the OCT data for optimization, TPEF images from the same location are presented before and after aberration correction in Fig. 2. Note that the OCT *en face* image presents the structural appearance of the sample, but is not of particularly high aesthetic quality due to the presence of large speckle and the comparatively low axial resolution. The main purpose of the OCT was to provide depth-resolved aberration correction and cross-sectional aiming of the focal plane in order to improve the MPM; therefore, having exquisite structural images was not important for our applications. However, the OCT image appearance could be improved by using a different light source that has a broader optical bandwidth, for example a titanium sapphire laser. After optimization, the OCT image in Fig. 2 had a ~22% increase in the sharpness image quality as defined by Equation 1. The TPEF image acquired after optimization is ~25% brighter and contains more detail in comparison to the images acquired before aberration correction. All of the images with AO ON and AO OFF were processed identically.

**Figure 3:**
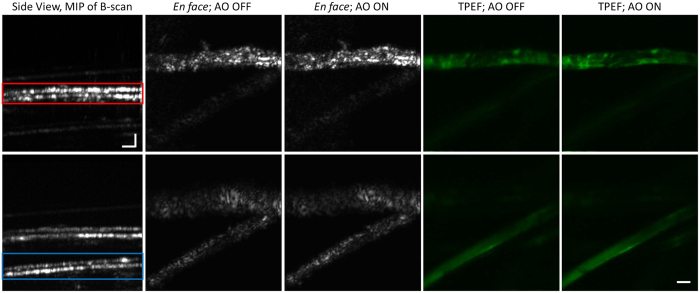
OCT and MPM images of two-layer phantom. The B-scan images were generated by maximum intensity projection (MIP) of the volume to generate a representative side-view of the sample to emphasize the axial locations of the two layers. The TPEF images in this figure are the average of 50 rigidly registered TPEF frames. Scale bar: 70 μm. Vertical scale bar for the B-scan: 70 μm.

In order to demonstrate our system’s depth resolved aberration correction capability, images of a two-layer phantom were acquired, with SAO correction performed on each of the layers independently. The two layers of the phantom were separated by ~100 μm as indicated by the blue and red boxes in the OCT B-scans on the left side of Fig. 3. The top row of Fig. 3 are images with SAO performed on the layer denoted by the red box, and the bottom row of images were acquired with SAO performed on the layer denoted by the blue box. Note that in this imaging experiment, prior to the SAO optimization, the defocus was first adjusted by the VFL, and then the SAO algorithm only optimized the astigmatism and coma terms. During optimization, the image metric was calculated on the maximum intensity projection of the layers within each box in Fig. 3. The *en face* OCT images presented are of all the layers to emphasize the shift of the focal position. The optimization performed on the layers in the red box had a ~14% increase in the sharpness metric of the *en face* OCT and a ~3% brightness increase in the TPEF image. The optimization performed on the layers in the blue box had a ~18% increase in the sharpness metric of the *en face* OCT and also a ~3% brightness increase in the TPEF image.

**Figure 4:**
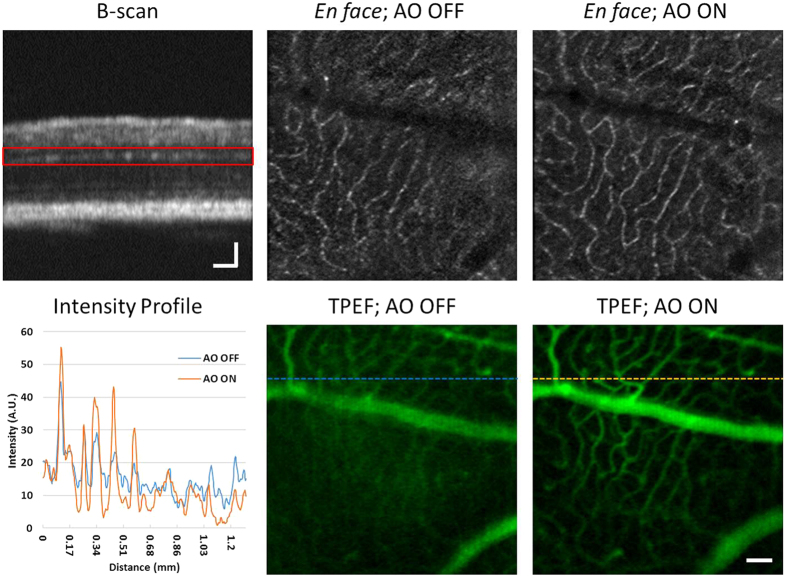
OCT (top row) and TPEF (bottom row, middle and right) images of the mouse retina before and after OCT-guided aberration correction. (Bottom left) The intensity profile of the TPEF images at the position of the dashed lines. The TPEF images in this figure are the average of 200 rigidly registered TPEF frames. Scale bars: 130 μm. Vertical Scale bar for B-scan: 40 μm.

Representative images of the mouse retinal vasculature acquired *in vivo* are presented in Fig. 4. The OCT images are presented on a linear scale, with the position of the focus within the retina indicated by the red box on the cross-sectional B-scan. The TPEF images acquired from that depth location are presented before and after OCT-guided SAO aberration correction. Qualitatively, the appearance of the retinal vessels is observed to be brighter and sharper following aberration correction in both OCT *en face* images and TPEF images. Quantitatively, the effect of SAO correction was demonstrated by comparing the signal intensity profile across the dotted lines (AO OFF, yellow; AO ON, blue) taken from the TPEF images.

**Figure 5:**
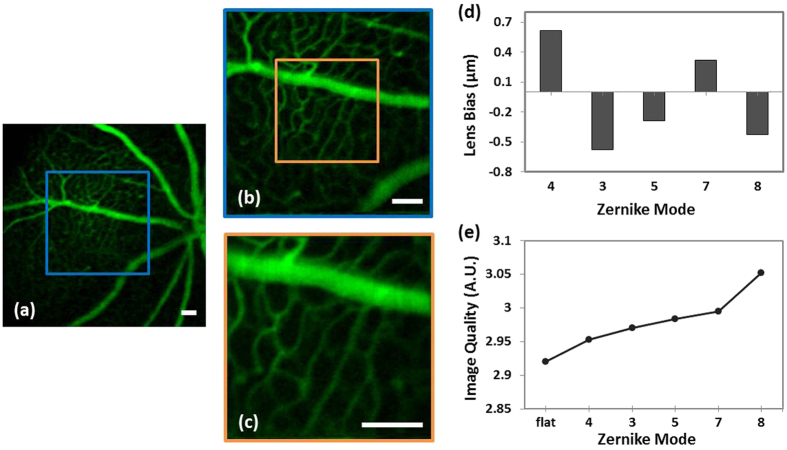
(**a**–**c**) TPEF images of mouse retina with different fields of view acquired after OCT-guided SAO optimization. (**d**) Relative bias of each Zernike mode applied to the MAL after the SAO optimization in Zernike modes. (**e**) Metric values (sharpness of the OCT *en face* images) during the SAO optimization. The TPEF images in this figure are the average of 200 rigidly registered TPEF frames. Scale bar: 200 μm.

Images with a different field of view acquired from the same imaging session are shown in Fig. 5(a–c). The OCT-guided SAO optimization was performed using the field of view of Fig. 5(c), then zoomed out to acquire TPEF images with larger field of view. Note that the image quality is best in the area of the isoplanatic patch where the aberration correction was performed, and that the image quality degrades with increasing distance from this region in the zoomed out images. Figure 5(d) shows the relative bias values of the Zernike modes applied to the multi-actuator adaptive lens after the image optimization. Figure 5(e) shows the metric values recorded from the *en face* OCT images after the optimization of each Zernike term, in which the ‘hill climbing’ effect of the SAO algorithm is clearly observed.

**Figure 6:**
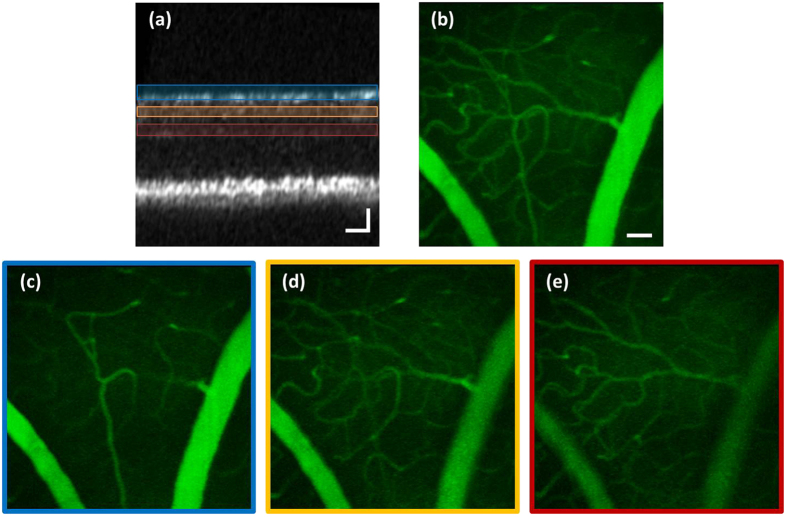
(**a**) OCT B-scan of mouse retina indicating the axial focus position by the blue, yellow and red boxes. (**b**) Maximum intensity projection of TPEF images of mouse retinal vasculatures after OCT-guided SAO optimization from the video frames in Video 1. (**c**–**e**) Mouse vasculature at different retinal layers after SAO aberration correction. The TPEF images in this figure are the average of 50 rigidly registered TPEF frames. Scale bar: 90 μm. Vertical Scale bar for B-scan: 40 μm.

TPEF images of vasculature at different layers of the mouse inner retina after OCT-guided SAO optimization are shown in Fig. 6(c,d). The corresponding axial focus positions are labeled in the OCT B-scan Fig. 6(a) by the blue (never fiber layer), yellow (inner plexiform layer) and red (outer plexiform layer) boxes. Video 1 demonstrates the axial sectioning capability of our MPM system, during this video, the axial focus was continuously shifted from the nerve fiber layer to the outer plexiform layer. Figure 6(b) is the maximum intensity projection of image frames in Video 1.

## Discussion

We have demonstrated a fast depth-resolved aberration correction method for MPM, and shown its ability to improve the TPEF image brightness and sharpness in static phantoms and for *in vivo* mouse retinal imaging. The aberration correction was performed using coherence-gated OCT images to guide an SAO algorithm. With the presented OCT system configuration, the optimization was performed in ~60 s, following which TPEF image acquisition was performed at ~100 ms per frame. For the imaging phantom, which was stationary, the time required to perform the optimization was not relevant. However, for *in vivo* imaging, in particular for retinal imaging in a mouse *in vivo*, a short optimization time was essential for reducing motion artifacts and limiting laser exposure to the tissues of the eye. Through successful optimization and improved image quality after aberration correction, we demonstrated that the motion observed during regular data acquisition with an anesthetized mouse, which is primarily due to breathing and heart beat artifacts, was not a limiting factor for the imaging performance. After successful optimization, we were able to navigate to and acquire aberration-corrected images of different regions of the retina.

Our SAO optimization time of ~60 s was limited by the speed of the OCT engine (spectrometer readout line rate), number of A-scans in a volume, and the number of points that were used in the optimization search algorithm for each Zernike mode. The optimization time could be readily reduced to several seconds or less by using a state-of-the-art custom spectrometer adjusted to the optical spectrum of the light source, or by reducing the size of the OCT volume during optimization and/or the number of points in the search algorithm. Furthermore, nonlinear multivariate optimization algorithms such as the one presented by Verstraete *et al*. can be explored to increase the converging rate and reduce the effect of non-orthogonality and fitting errors of the Zernike modes produced by the optical deformable element^34^.

The impact of performing the aberration correction using a single photon process, and in particular from the same light source that is used for MPM excitation, is evidenced through the strength of the OCT signal used as the merit function and the corresponding time required for optimization. With the bright OCT images, the optimization can be performed from a single acquisition at each step of a Zernike mode coefficient value. In contrast, due to the low signal strength of the TPEF, multiple images would need to be acquired and averaged in order to generate a single data point, making the time needed to run the optimization algorithm on the order of minutes^26^. Moreover, due to the greater sensitivity of OCT detection in comparison to TPEF, the OCT-based optimization can in general be performed at a lower incident power, thereby reducing the overall laser exposure to the sample, in this case the delicate retinal tissues.

An important benefit of using coherence-gated SAO is that a specific depth layer can be selected for optimization. For the case of the imaging phantom, the aberration correction could be repeated at different depths, as was demonstrated in Fig. 3. This is even more important for multi-layer tissue samples, such as the retina, that have bands of strongly scattering tissues, and aberrations that change as a function of depth. With the depth-resolved OCT data guiding the SAO optimization, the *en face* images used for the merit function can be extracted from the specific layer of interest without influence from the other retinal layers.

Alternative approaches to coherence-gated aberration correction have been proposed in the literature. The combination of coherence-gated wavefront sensing with two-photon microscopy was reported using a virtual Shack-Hartmann sensor^35^. Alternative approaches to coherence-gated wavefront sensing using a virtual Shack-Hartmann sensor have also been reported^36^,^37^. The combination of coherence-gated detection with a lenslet-based Shack-Hartmann wavefront sensor was reported as well^38^,^39^. A limitation of these methods is the added optical and electronics hardware complexity required for implementing the wavefront measurement. Another limitation of these coherence-gated wavefront measurement techniques are that they do not provide real-time visual feedback on where in the sample the focal waist was positioned.

In this report, a transmissive multi-actuator adaptive lens (MAL) was used as the aberration correcting element. The methods presented for improving the TPEF signal based on OCT-guided SAO could also be used with other adaptive elements, such as deformable mirrors. Adaptive elements are commonly placed at planes that are optically conjugated to the pupil; this is accomplished using relay lenses, which increases the physical size of the system and the complexity of integration with existing microscopes. For imaging configurations that use an objective lens, the MAL can be positioned adjacent to the back aperture of the objective, simplifying the integration with existing systems^22^.

The successful demonstration of the MAL for MPM optimization using the OCT-guided SAO is encouraging for small animal retinal imaging. Although the laser power incident on the eye was higher than the ANSI recommended limits, the optical intensity was in line with other works that report no physiological changes at the retina^9^. With a few changes, in particular decreasing the incident laser power, the OCT-guided approach to SAO described in this report could be also be developed and applied for aberration corrected MPM in human retina.

## Additional Information

**How to cite this article**: Cua, M. *et al*. Coherence-Gated Sensorless Adaptive Optics Multiphoton Retinal Imaging. *Sci. Rep.* **6**, 32223; doi: 10.1038/srep32223 (2016).

## Supplementary Material

Supplementary Information

Supplementary Video 1
