# Supplementary material for: Coherence-Gated Sensorless Adaptive Optics Multiphoton Retinal Imaging
Source: Sci Rep. 2016 Sep 7;6:32223. doi: 10.1038/srep32223 (PMC5013266; doi:10.1038/srep32223)
Supplement: Supplementary Information [file srep32223-s1.pdf]

Coherence-Gated Sensorless Adaptive Optics Multiphoton Retinal Imaging

Michelle Cua[a ‡], Daniel J. Wahl[a ‡], Yuan Zhao[a], Sujin Lee[a], Stefano Bonora[b],
Robert J. Zawadzki[c,d], Yifan Jian[a, *], Marinko V. Sarunic[a, *, 1]
[a] School of Engineering Science, Simon Fraser University, Burnaby, BC V5A 1S6 Canada
[b] CNR-Institute for Photonics and Nanotechnology, Via Trasea 7, 35131, Padova, Italy
[c] UC Davis RISE Small Animal Ocular Imaging Facility, Department of Cell Biology and Human Anatomy, University of California Davis, Davis, CA 95616, USA
[d] Vision Science and Advanced Retinal Imaging laboratory (VSRI), Department of Ophthalmology & Vision Science, University of California Davis, Sacramento, CA 95817 USA
[‡] These authors contributed equally.
[*] These authors are co-senior authors.
[1] Send correspondence to: Yifan Jian, yjian@sfu.ca; and Marinko V. Sarunic, msarunic@sfu.ca.

Video 1 TPEF images of mouse retinal vasculature with the axial focus continuously shifted from the nerve fiber layer to the outer plexiform layer.
